# The Influence of Dietary Fat on Liver Fat Accumulation

**DOI:** 10.3390/nu6115018

**Published:** 2014-11-10

**Authors:** Charlotte J. Green, Leanne Hodson

**Affiliations:** Oxford Centre for Diabetes, Endocrinology and Metabolism (OCDEM), Churchill Hospital, University of Oxford, Oxford OX3 7LE, UK; E-Mail: charlotte.green@ocdem.ox.ac.uk

**Keywords:** dietary fat, liver, steatosis, NAFLD, VLDL-TG, human

## Abstract

Obesity is a known risk factor for the development of non-alcoholic fatty liver disease (NAFLD); however, it has been suggested that dietary fat, both amount and composition, may play a pivotal role in its development, independent of body fatness. Studies that have investigated the role of dietary fat on liver fat accumulation are reasonably sparse. We review here the available work that has investigated the impact of dietary fat: amount, composition and frequency, on liver fat accumulation in human observational and intervention studies. Overall, it would seem that total calorie consumption, rather than dietary fat composition, is an important factor in the development of fatty liver disease in humans.

## 1. Introduction

The prevalence of obesity and the metabolic syndrome are increasing worldwide in young and older individuals. The deposition of fatty acids in non-adipose tissues (ectopic fat) is thought to be an important factor in the development of obesity-related metabolic abnormalities. Adipose tissue plays a crucial role in “buffering” the flux of lipids in systemic circulation during the postprandial period; however, when the buffering capacity of adipose tissue is impaired, then other tissues, such as skeletal muscle and liver are exposed to excess lipids [1]. The liver plays a major role in metabolic regulation of dietary nutrients including fat and carbohydrates. In health the liver rapidly adapts to altered nutrient fluxes that occur from a fasted to fed state. The accumulation of intrahepatic fat is now recognized as a contributor to the pathology of metabolic diseases [2]. Why the liver starts to store fat is not well understood. It has been proposed that when fatty acids exceed the liver’s capacity for removal (*i.e.*, via secretion or oxidation pathways) they are stored as triglyceride (TG) [3,4,5]. A net retention of TG is the prerequisite for the development of non-alcoholic fatty liver disease (NAFLD) [6].

Obesity is a well-documented risk factor for NAFLD, which is one of the most common liver diseases in developed countries [7,8]. NAFLD occurs in individuals who do not consume large amounts of alcohol (greater than 30 and 20 g of alcohol daily for men and women respectively) [8,9]. The prevalence of NAFLD is reported to occur in ~70% of those with type 2 diabetes [10] and between 6% and 51% of the general population, depending on the assessment method used [11]. NAFLD is not a single disease but rather encompasses a spectrum of conditions: simple fatty liver (hepatic steatosis), more severe steatosis coupled with necroinflammation with or without fibrosis (non-alcoholic steatohepatitis (NASH)), to severe liver disease such as cirrhosis and potentially hepatocellular carcinoma (HCC) [8,9,10,12,13]. Unlike steatosis, NASH indicates the progression of liver disease and has been reported to confer an approximately two-fold higher mortality than simple steatosis, largely accounted for by liver-related complications [12,13]. The progression from NASH to cirrhosis and HCC will only occur in a minority of NAFLD patients [14]. NAFLD is associated with increased risk of all-cause and liver-related mortality and increased risk of cancer, kidney disease and cardiovascular disease (CVD) independent of age, gender and smoking [15,16]. Moreover, insulin resistance and low-grade inflammation associated with NASH may play a role in the development of HCC in a minority of genetically pre-disposed patients [16].

## 2. Quantifying Hepatic Steatosis

Intrahepatic TG can be measured by a number of methods: chemical, histological and imaging modalities. For the majority of methods, hepatic steatosis is defined when intrahepatic TG content exceeds 5% of hepatic tissue or hepatocytes [17,18,19]. The methods for assessing intrahepatic fat content have been well reviewed [17]. In the majority of cases the use of histology to assess intracellular TG provides semi-quantitative and qualitative information [20]. For example, the size of the lipid droplets (*i.e.*, steatotic pattern) and the proportion of hepatocytes containing lipid can be measured. The steatotic pattern is defined as either high-grade microvesicular steatosis, consisting of fatty vesicles measuring less than 1 µm filling the hepatocyte cytoplasm, where the nucleus remains located centrally [21,22]. Alternatively, macrovesicular steatosis consists of one large vacuole of fat, which displaces the nucleus to the periphery of the hepatocyte [21,22]. Currently, liver biopsy is considered the gold standard for diagnosing and grading steatosis. Biopsies are graded on a scale from 0 to 3 where 0 is considered normal (*i.e.*, up to 5% of cells affected) and 3 is severe (*i.e.*, ≥67% cells affected) [23]. It has been reported that pathological grading of histological sections is subject to inter- and intra-observer variation when assessing histological features [20]. Recently, Pournik *et al.* [24] reported high inter-observer agreement using the NAFLD activity score (NAS). Additionally, as single biopsies carry significant risk of sampling variability, it has been suggested that two or more core samples give better diagnostic yield [25]. The biopsy procedure is invasive (and impractical to do in large numbers and in healthy controls), semi-quantitative, prone to sampling error and not sensitive enough to detect small changes in steatosis; therefore, imaging modalities, in particular ultrasound and magnetic resonance imaging (MRI) with or without spectroscopy (MRS) are becoming more frequently utilised. The sensitivity of these methods varies, with MRS (and more recently developed MRI techniques) accurately quantifying hepatic fat content whilst ultrasound does not [17,23].

## 3. Liver Fatty Acid Metabolism: Transitioning from the Fasted to Fed State

**Figure 1:**
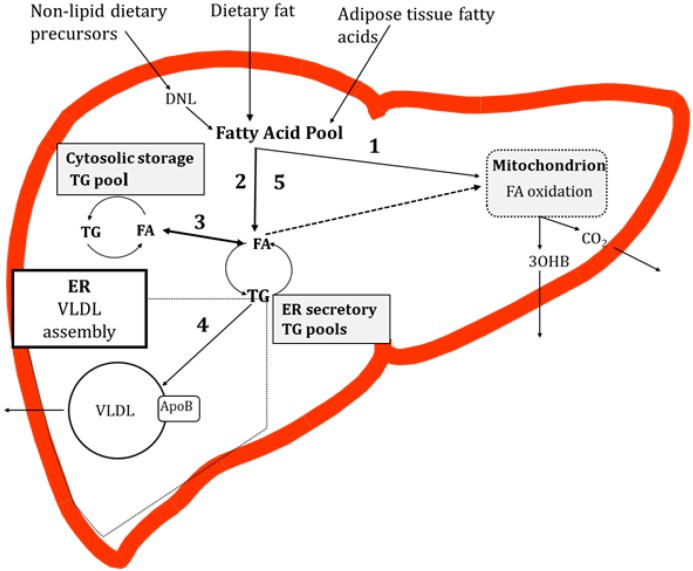
Overview of hepatic fatty acid metabolism in the postprandial state. Fatty acids enter a pool where they may be partitioned into oxidation (1) or esterification (2) pathways. There are TG storage and secretory pools. Fatty acids liberated from the hydrolysis of TG in the secretory TG pool, or TG particles, may then be partitioned to a storage TG pool (3). TG in the secretory pool is utilised for very low-density lipoprotein (VLDL) production (4) which enters systemic circulation. It remains unclear if fatty acids liberated from the TG pools enter oxidation pathways (dotted line, (5)). Abbreviations: TG, triglyceride; VLDL, very low-density lipoprotein; DNL, *de novo* lipogenesis; FA, fatty acid; NEFA, non-esterified fatty acids; ApoB, apolipoprotein B; 3OHB, 3-hydroxybutyrate; ER, endoplasmic reticulum.

In the fasting state non-esterified fatty acids (NEFAs), derived from the intracellular lipolysis of adipose TG (subcutaneous and visceral), enter the liver and are a primary substrate for fatty acid oxidation and a precursor for hepatic TG synthesis [26]. In combination with the fatty acids derived from the lipolysis of adipose tissue, fatty acids synthesised via the intrahepatic *de novo* lipogenesis (DNL) pathway and those already present in the cytosol may be utilised for very low-density lipoprotein (VLDL)-TG production [27]. Work in rodent hepatocytes has demonstrated that the majority of fatty acids taken up by the liver are channeled into a common pool [28,29] before being directed to other fates. In the fed state, dietary (exogenous) fatty acids enter the liver and mix with fatty acids already present in the common pool. From here fatty acids are partitioned into esterification or oxidation pathways. If fatty acids are esterified, then the resulting TG accumulates in both the cytosol (storage pool), and in the endoplasmic reticulum (ER) membrane and ER lumen (secretory pools), each pool having a distinct rate of turnover [30,31]. Some of the fatty acids that reach the secretory TG pools are derived from the mobilization of stored TG [30,31,32]. To our knowledge, studies in human hepatocytes demonstrating fatty acid flux through TG pools have yet to be reported. TG in the secretory pool is a substrate for the formation of VLDL, which, once lipidated, is secreted from the liver into systemic circulation (Figure 1).

## 4. Potential Causes of Hepatic TG Accumulation

The accumulation of liver TG represents a potential imbalance between pathways of fatty acid input and removal. As humans spend the majority of the day in a postprandial state [33,34] and typically consume a fat rich diet (35% total energy (TE) [35]), it could be speculated that dietary fatty acids play an important role in liver TG metabolism. Along with dietary factors, which would include not only the amount but also the composition of nutrients consumed, lifestyle factors may play a role. For example, a sedentary lifestyle may also contribute to the development on hepatic steatosis [36]. In addition to dietary fat, the amount and type of carbohydrate consumed may also play an important role in the development of hepatic steatosis; although of interest it is outside the scope of this review and reviewed by Moore *et al.* in this edition. Furthermore, dietary cholesterol has been strongly associated with risk of NAFLD development in obese adults and children [37,38] and a high intake of dietary cholesterol has been reported to be an independent predictor of cirrhosis development [39]. The mechanisms by which cellular cholesterol induces liver injury may be related to altered cholesterol homeostasis and toxicity due to cellular cholesterol overload [12]. Despite the potential causes of hepatic steatosis being extensively reviewed [10,14,40,41,42,43,44,45,46,47], surprisingly few have discussed the role of dietary fat. Therefore, the focus of this review is on the impact that dietary fat, in both amount and composition, has on the development of hepatic steatosis in humans.

### 4.1. Contribution of Specific Fatty Acids to Liver TG

Determining the contribution of specific fatty acid sources (Figure 1) can be achieved with the use of stable-isotope tracers. Using a multi-tracer approach Donnelly *et al.* [48] determined the contribution of specific sources of fatty acid to liver and VLDL-TG in NAFLD patients (*n* = 8). After five days of labeling, they reported there was no difference in the contribution of fatty acids originating from systemic NEFA, DNL or diet to liver and VLDL-TG [48]. On the basis of this observation, the authors suggested that VLDL-TG may be used as a surrogate marker of the liver TG/fatty acid pool [48]. Dietary fatty acids have been reported to contribute 2%–28% of VLDL-TG [48,49,50,51]. Fatty acids originating from systemic NEFA contribute 45%–75% and from hepatic DNL fatty acids contribute 13%–37% to VLDL-TG [48,49,50,51]. The wide-range in findings may be explained by differences in the length of the postprandial phase, the type of test meal fed and hepatic uptake, and/or alterations in the turnover time of the hepatic TG pool, which may be influenced by size of the pool.

### 4.2. Trafficking of Dietary Fatty Acids to Liver TG

The trafficking of dietary fat into liver TG has been assessed using ^13^C MRS [52]. Individuals with diet-controlled type 2 diabetes (T2D) had significantly more liver TG than age- and BMI-matched controls (121 *vs.* 48 mmol/L, respectively) [52]. To investigate dietary fatty acid trafficking though the liver, participants consumed a mixed test meal (28 g fat which had ^13^C tracer incorporated) and then 5 h later consumed an “unlabeled” test meal. Incorporation of ^13^C from meal fat into liver TG occurred more rapidly and to a greater extent in the individuals with T2D than the controls (peak incorporation 4 *vs.* 6 h and peak uptake ~13% *vs.* ~9% of ingested meal fat, respectively) [52]. The appearance of ^13^C meal fatty acids in TG-rich lipoproteins was also rapid with peak incorporation being achieved earlier in the T2D (6 h) compared to the control (8 h) group. After consumption of the second meal, liver TG ^13^C enrichment declined, whilst the appearance of ^13^C in TG-rich lipoproteins increased in both groups. The authors suggested these rapid fluxes of fatty acids in and then out of the liver during the postprandial period protect the body from excessive plasma TG fluxes in the immediate postprandial period [52]. An alternative way to view these data is that the rapid and more pronounced appearance of dietary fatty acids in liver TG in the T2D group is due to the buffering capacity of adipose tissue potentially being impaired, thus more dietary fat is “spilling over” to the liver leading to greater TG accumulation [1]. This work highlights how rapidly dietary fatty acids reach the liver; if fat- containing foods are being consumed frequently then there will be a constant flux of meal fatty acids through the liver, which may exceed the secretion (as VLDL) and/or oxidation pathway capacities.

## 5. Associations between Dietary Fat and Liver TG

Only a handful of studies have investigated the association between liver fat content and dietary fat intake [53,54,55,56]. Koch *et al.* [53] investigated the association between dietary pattern, assessed by food frequency questionnaire (FFQ), and liver fat content measured by MR imaging in 354 adults from the PopGen control cohort. They found that intake of “other fats” and cheese was not associated with liver fat whilst alcohol was [53]. In contrast, Mollard and colleagues [55] explored the role of dietary fat (assessed by FFQ) as a determinant of hepatic steatosis (measured by MRS) in 74 overweight adolescents (aged ~15 years). In this cohort 39 individuals had liver fat <5.5% and 29 had liver fat ≥5.5%. Results showed a greater proportion of adolescents with liver fat ≥5.5% consumed >35% TE as fat and more fried food compared to adolescents with liver fat <5.5% [55]. Total fat intake was significantly associated with hepatic TG, even after adjustment for confounding variables. Using regression analysis it was demonstrated that a fat intake of >35% TE was a significant predictor of liver TG (11.76 (1.60, 86.62)) odds ratio (95% confidence interval) in the model adjusted for all confounding factors, whilst intakes of saturated fat >10% TE was not [55]. A cross-sectional study investigated the association between acute (1 day) and habitual (10 day) dietary intake and liver fat, measured using computed tomography (CT), in 42 abdominally-obese men [54]. There was no association between liver fat and acute dietary fat intakes (mean intake 29.9% TE) but a positive association (*r* = 0.40, *p* < 0.010) was found between liver fat and habitual fat intakes (mean intake 31% TE) [54]. Given the day to day variation in eating patterns, these data suggest that for dietary fat to have an effect on liver fat accumulation, habitual intake is important. A study in an Indian population compared individuals with ultra-sound determined NAFLD (cases, *n* = 98) to those without (controls, *n* = 102) to determine nutritional risk factors that may contribute to liver TG [56]. A semi-quantitative FFQ was utilised to assess dietary intakes. Although the groups were matched for age and sex, cases had a significantly higher BMI and waist circumference and consumed significantly more fat, carbohydrate and protein than controls [56]. In a stepwise regression model, BMI, waist circumference and percent fat intake were independent predictors of hepatic steatosis [56]. However, caution is required due to the challenges of accurately assessing dietary intakes [57]. The majority of studies have used FFQs which offer the advantage of estimating foods habitually consumed. However, they have known limitations including: memory of the respondent, incorrect estimation of portion sizes, coding and computation error, and food composition databases being incomplete and not up to date [57,58,59]. Furthermore, these studies have used a variety of techniques to quantify the amount of liver fat, which makes it challenging to compare findings across studies. Taken together, the evidence from epidemiological studies would suggest that increased intakes of dietary fat are related to the risk of developing hepatic steatosis. However, as a high fat diet is typically a high energy diet, consideration is needed when interpreting these data. Thus, more work is needed to confirm the relationship between dietary fat and hepatic steatosis.

## 6. Intervention Studies: Evidence for Dietary Fat Altering Liver Fat Content

Only a small number of studies have undertaken interventions to investigate the effect of acute and chronic changes in the amount and composition of dietary fat on liver fat accumulation (Table 1) [60,61,62,63,64,65,66,67,68,69,70,71]. Studies that have been undertaken have used diets that were iso-, hypo- and hyper-caloric, in a wide range of subjects in terms of adiposity and age (Table 1).

**Table 1:** Overview of intervention studies that have investigated the effect of dietary fat on liver fat content.

Ref	Subjects	Dsn	Lngt	Eng	Diet	Fat (%TE)	Measure Liver Fat	Baseline Liver Fat (%)	Change Liver Fat (%)
[70]	10 F	X	2 wk	Iso	LF	16% Tot	MRS	10	↓20
	BMI 33				HF	56% Tot			↑35
	Age 43								
[67]	7 M/13 F	P	4 wk	Iso	LF, low SFA, low GI	23% Tot	MRS	2.2 ^†^	↓0.44
	BMI 26.9					7% SFA			
	Age 69								
	6 M/9 F				HF, high SFA, high GI	43% Tot		1.2 ^†^	↑0.001
	BMI 28.1					24% SFA			
	Age 69								
[69]	20 M	P	3 wk	Iso	LF *vs.* HF	20% Tot	MRS	2.2	↓13
	BMI 29					55% Tot			↑17
	Age 34								
[61]	37M/8F	P	8 wk	Iso	MUFA −ex	42% Tot; 7% SFA; 5% PUFA; 27% MUFA −ex	MRS	7.4	↓30
	T2D								
	BMI 30				MUFA +ex	42% Tot; 7% SFA; 5% PUFA; 16% MUFA +ex		11.6	↓22
	Age 35–70 ^‡^								
[60]	67M/F	P	10 wk	Iso	SFA *vs.* *n*-6 PUFA	~42% Tot, ~20% SFA, ~4% PUFA	MRS	3.2	↑8
	BMI 30.5					~39% Tot, ~10% SFA, ~13% PUFA			↓26
	Age 30–65 y ^‡^								
[62]	5 M/13 F	P	2 wk	Hypo	LC	34% Tot	MRS	19	↓26
	BMI 35				LCHO	59% Tot		22	↓55
	Age 45 y								
[63]	35 M/135 F	P	6 m	Hypo	LCHO	30% Tot	MRS	7.6	↓47
	BMI 32				LF	≤20% Tot		9.6	↓42
	Age 45 y								
[64]	4 M/18 F	P	11 wk	Hypo	LF *vs.* HF	20% Tot	MRS	11.2	↓>45 ^§^
	BMI 37					75% Tot		12.4	↓>35 ^§^
	Age 44 y								
[71]	9 M/17 F	I	7 m	Hypo	LC	30% Tot	MRS	10.8	↓28
	BMI 32.4					10% SFA			
						10% MUFA			
	Age 52y					10% PUFA			
[66]	39 M	P	4 d	Hyper	HF	60% Tot, 28% SFA	MRS	~11 ^§^	↑86
	BMI 23				HF/HFrc	60% Tot, 3.5g Frc/kg FFM		~12 ^§^	↑133
	Age 24 y								
[68]	15 M	I	3 d	Hyper	HEHF	69% Tot	MRS	2.01	↑112
	BMI 23.4								
	Age 25 y								
[65]	41 M/F	P	7 wk	Hyper	SFA	37% Tot, 17% SFA, 5% PUFA	MRI	0.96	↑58
	BMI 18–27 ^‡^				*n*-6 PUFA	40% Tot, SFA 11%, PUFA 13%		0.75	↑5
	Age 20–38 y ^‡^								

Mean data from paper unless otherwise stated. Abbreviations: Ref, reference; Dsn, design; Lngt, length of study; Eng, energy intake; %TE, percentage of total energy; M, males; F, females; BMI, body mass index (kg/m^2^); y, years; P, parallel; X, cross-section; I, intervention; wk, week; m, month; d, day; Iso, iso-caloric; Hypo, hypo-caloric; Hyper, hyper-caloric; LF, low fat; HF, high fat; GI, glycaemic index; T2D, type 2 diabetes; FA, saturated fat, MUFA, monounsaturated fat; PUFA, polyunsaturated fat; Tot, total; +/−, with or without; ex, exercise; MRS, magnetic resonance spectroscopy; MRI, magnetic resonance imaging; ↑, increase; ↓, decrease; LC, low calorie; LCHO, low carbohydrate; HF/HFrc, high fat, high fructose; Frc, fructose; FFM, fat free mass; HEHF, high energy, high fat; ^†^ median; ^‡^ range; ^§^ estimated from graph.

### 6.1. Iso-Caloric Diets

To date, five studies have been undertaken using iso-caloric diets, three comparing low *vs.* high fat [67,69,70] and two investigating the effect of specific fatty acids [60,61]. Consumption of a low fat diet (total fat <25% TE) appears to lead to a decrease in liver fat compared to consumption of a high fat diet (total fat <40% TE) (Table 1). Utzschneider *et al.* noted [67] minimal change in liver fat content after consumption of either a diet with a high-total and saturated fat (SFA) content or a low amount of total and SFA. Why the changes were not as striking as those of van Herpen *et al.* [69] and Westerbacka *et al.* [70] is unclear but may be related to the age of the subjects, differences in the amount of total fat, length of study, or the possibility that subjects were already consuming a habitual diet that was not so dissimilar to the intervention diet.

The two studies that have investigated the effects of specific fatty acids on liver fat accumulation focused on monounsaturated fat (MUFA), with and without exercise [61], or the effect of a diet enriched with either SFA or *n*-6 polyunsaturated (*n*-6 PUFA) fat [60]. Consumption of a diet rich in MUFA (27% TE) lowered liver fat to a greater degree than consumption of a diet containing 16% MUFA with prescribed aerobic exercise (two sessions per week). The authors suggested the MUFA-enriched diet may have lowered liver fat by positively influencing adipose tissue cross-talk with liver metabolism via regulation of inflammatory marker synthesis and adipokines [61]. Consumption of a diet enriched with *n*-6 PUFA for 10 weeks notably decreased liver fat compared to the SFA enriched diet, which increased liver fat in obese participants with low amounts (<5%) of liver fat [60]. The authors speculated the mechanism responsible for the decrease in liver fat content on the *n*-6 PUFA diet was due to PUFAs preferentially undergoing β-oxidation, compared to SFAs; also that PUFA inhibits *de novo* hepatic fatty acid synthesis and lipogenic gene expression [60]. An alternative possibility is that as PUFAs are preferentially partitioned toward blood phospholipid fractions they are not available for TG formation [72].

### 6.2. Hypo-Caloric Diets

There have been four studies that have investigated the effect of diet composition on liver fat in subjects who consumed a hypo-caloric diet [62,63,64,71] (Table 1). Typically participants in these studies have consumed approx. half of the recommended daily energy intake for men and women, for periods between 2 weeks and 7 months. Body weight decreased on these diets by around 6% [62,63,64,71]. In general, regardless of the total amount or composition of fat consumed, a hypo-caloric diet resulted in a notable decrease in liver fat in short and longer term studies (Table 1). Bian *et al.* [71] noted that liver volume, along with fat, significantly decreased. These data demonstrate that the most important contributing factor to whether liver fat accumulation occurs is the amount of total energy consumed, rather than the composition of the diet. Notably, liver fat decreases more rapidly when a hypo-caloric diet devoid of carbohydrate is consumed, compared to calorie restriction alone [62,64].

### 6.3. Hyper-Caloric Diets

Three studies have investigated the influence of overfeeding acutely (3–4 days) or longer-term (7 weeks) on liver fat accumulation [65,66,68] (Table 1). Liver fat increased to a significant extent (between 86% and 133%) in acute overfeeding studies; notably, participants in both studies were young, healthy males [66,68]. Although the increase in energy was not reported by Sobrecases *et al.* [66] they demonstrated that feeding a fat-enriched diet (~60%TE) increased liver fat, an effect that was exacerbated when fructose was also consumed. Doubling habitual calorie intake (69% TE as fat) for 3 days resulted in a 112% increase in liver fat in individuals with low baseline liver fat and without weight gain [68]. Rosqvist *et al.* [65] investigated the effect of hyper-caloric feeding of either SFA or *n*-6 PUFA on liver fat accumulation in young, healthy adults with very low amounts of liver fat at baseline (Table 1). They found that 7 weeks of overfeeding with SFA increased liver fat to a greater extent than overfeeding with n-6 PUFA, independent of weight gain (~2.5% in each group). Although liver fat increased dramatically in studies by van der Meer *et al.* [68] and Rosqvist *et al.* [65], subjects remained well below the cut-off of hepatic steatosis (5%) due to the very low levels at baseline. Based on the findings of Sobrecases *et al.* [66], it appears that liver fat markedly increases in response to high-energy, high-fat feeding in subjects with steatosis to a similar extent as those with low baseline liver fat levels. Taken together, these data clearly demonstrate that excessive calorie consumption for short and longer periods increase liver fat irrespective of increases in body weight. The lower accumulation of liver fat on the n-6 PUFA compared to the SFA diet maybe due to differences in the metabolism and partitioning of the fatty acids within the liver, as discussed above.

### 6.4. Supplementation Studies

Only a few studies have been undertaken looking at the effect of supplementation with long-chain *n*-3 fatty acids on liver fat content [73,74,75]. Consumption of a high amount of eicosapentaenoic acid (EPA) (1080–1840 mg) and docosahexaenoic acid (DHA) (1520–2240 mg) for 8 weeks or 15–18 months resulted in a decrease in liver fat between 19% and 29% [73,74]. Cussons *et al.* [73] reported that liver fat decreased only when individuals started with a higher amount, that is >5% of liver fat. As Scorletti *et al.* [74] only studied individuals with NAFLD, discrimination between effects of n-3 fatty acids on high and low liver fat cannot be made. In contrast, Vega *et al.* [75] reported no change in liver fat content after consumption of fish oil for 8 weeks. The discrepancy in findings between studies may be due to differences in supplementation fatty acid composition, the amount of n-3 fatty acids consumed or differences in participant characteristics.

## 7. Dietary Fat Alters Liver Fat: Potential Mechanisms

Why liver fat starts to accumulate when individuals are metabolically healthy may be due to the composition of their diet. For example, with iso-caloric feeding, having a diet rich in fat, notably SFA, caused TG accumulation, whereas diets rich in MUFA or PUFA tended to decrease liver TG. The mechanisms that may play a role is the potentially lipotoxic effects of SFAs, including ER stress, oxidative stress, and mitochondrial dysfunction [76,77]. Increasing evidence suggests ER stress is associated with SFA induced cellular dysfunction; NAFLD patients have been reported to have increased levels of ER stress markers [76]. The mechanism by which SFA induce ER stress is unclear but recent evidence suggests disordered phospholipid metabolism may be a key factor [76]. Additionally, the accumulation of liver fat when consuming a diet enriched with SFA may be due to SFAs up-regulating lipogenic genes such as stearoyl-CoA desaturase (SCD) which promote TG formation [78]. In human liver cell lines (HepG2, Huh 7) culturing with palmitic acid resulted in increased apoptosis whilst culturing with a higher concentration of oleic acid caused greater steatosis without apoptosis [79]. Using a lipidomic approach it was demonstrated that in total plasma and hepatic lipids the concentration of saturated and monounsaturated fatty acids tended to be higher in individuals with fatty liver compared to healthy controls [80,81]. Differences in the concentration of saturated and monounsaturated fatty acids in plasma and hepatic total lipids between control subjects and individuals with NASH did not tend to go in the same direction [80,81]. Interpreting the findings from these studies is challenging and may be due to differences in dietary intake between the groups, alterations in endogenous metabolism of fatty acids and/or the amount and composition of total lipid in plasma or liver. Any reported differences in fatty acid composition when measuring total plasma lipids may not provide an accurate representation due to differences in lipoprotein profiles [59,82]. The synthesis of TG within the liver may be advantageous, as an accumulation of lipid intermediates such as diacylglycerol and acylcarnitines is associated with insulin-resistance and inflammation [76,83].

Disentangling the effect of weight gain due to excess calories and the effect of dietary composition is challenging. It is plausible that consumption of a hyper-caloric diet rich in fat results in an increased flux of fatty acids to the liver that exceeds the capacity for secretion (as VLDL-TG) or oxidation. In line with this, Koopman *et al.* [84] reported liver fat increased to a notably greater extent when the same amount of calories were consumed more frequently, that is, three meals a day with snacks verses three meals only.

The noted reductions in liver fat with hypo-caloric diets, increased MUFA or *n*-6 PUFA intakes, or supplementation with *n*-3 fatty acids, may be the result of multiple mechanisms. For example, although hypo-caloric diets may contain a high proportion of fat, it is likely that relative to iso- or hyper-caloric diets rich in fat, total fat intakes will be attenuated, resulting in a decreased flux of dietary fat to the liver. For diets rich it MUFA and/or *n*-6 PUFA it is plausible that these fatty acids are preferentially partitioned toward oxidation pathways compared to SFAs [85,86]. Furthermore, PUFAs suppress the expression of lipogenic genes [78] which may result in the partitioning to lipid pools other than TG [72].

## 8. Conclusions

Evidence for dietary fat to influence liver TG accumulation is relatively sparse. Given the evidence presented here, it would appear that total calories consumed, rather than dietary composition, is a contributing factor in the development of fatty liver disease. However, consumption of an iso-caloric diet rich in SFA may be an important determinant of liver fat accumulation; the exact mechanisms are yet to be elucidated. In contrast, a diet rich in PUFA (*n*-6 or *n*-3) appears to have a negative effect on the degree of TG that accumulates within the liver. It could be speculated that changes in dietary intake will not completely mirror changes in hepatic fatty acid metabolism due to the complex association between adiposity, insulin resistance, and genetic predisposition. Therefore, more work is required to disentangle the individual components that influence the development of NAFLD. On the basis of the small amount of studies undertaken it would seem logical to recommend an iso-caloric diet (if weight loss is not a goal) of moderate total fat intake and low (<10%TE) in SFA to lower the risk of NAFLD development.
